# Resistance to Obesity in SOD1 Deficient Mice with a High-Fat/High-Sucrose Diet

**DOI:** 10.3390/antiox11071403

**Published:** 2022-07-19

**Authors:** Atsushi Sato, Yasunaga Shiraishi, Toyokazu Kimura, Ayumu Osaki, Kazuki Kagami, Yasuo Ido, Takeshi Adachi

**Affiliations:** 1 Department of Internal Medicine, Division of Cardiovascular Medicine, National Defense Medical College, 3-2 Namiki, Tokorozawa 359-8513, Saitama, Japan; atsushi19821005@yahoo.co.jp (A.S.); oyotikuuyakim@gmail.com (T.K.); ayumu.osaki@gmail.com (A.O.); mirror.1028k@gmail.com (K.K.); yasaoido@me.com (Y.I.); 2 Division of Environmental Medicine, National Defense Medical College Research Institute, 3-2 Namiki, Tokorozawa 359-8513, Saitama, Japan; sirayasu10@hotmail.com

**Keywords:** SOD1, metabolic syndrome, superoxide anion, ATP, mitochondrial intermembrane space, insulin secretion, AMPK, oxygen consumption

## Abstract

Metabolic syndrome (Mets) is an important condition because it may cause stroke and heart disease in the future. Reactive oxygen species (ROSs) influence the pathogenesis of Mets; however, the types of ROSs and their localization remain largely unknown. In this study, we investigated the effects of SOD1, which localize to the cytoplasm and mitochondrial intermembrane space and metabolize superoxide anion, on Mets using SOD1 deficient mice (SOD1^−/−^). SOD1^−/−^ fed on a high-fat/high-sucrose diet (HFHSD) for 24 weeks showed reduced body weight gain and adipose tissue size compared to wild-type mice (WT). Insulin secretion was dramatically decreased in SOD1^−/−^ fed on HFHSD even though blood glucose levels were similar to WT. Ambulatory oxygen consumption was accelerated in SOD1^−/−^ with HFHSD; however, ATP levels of skeletal muscle were somewhat reduced compared to WT. Reflecting the reduced ATP, the expression of phosphorylated AMPK (Thr 172) was more robust in SOD1^−/−^. SOD1 is involved in the ATP production mechanism in mitochondria and may contribute to visceral fat accumulation by causing insulin secretion and insulin resistance.

## 1. Introduction

Metabolic syndrome (Mets) is a condition in which visceral obesity combined with hypertension, hyperglycemia, and abnormal lipid metabolism predisposes to cardiovascular disease and is closely related to insulin resistance [1]. Mets is a complex syndrome because it is related to many organs, including the heart, liver, skeletal muscle, and adipose tissue, and the exact pathogenesis is still largely unknown [2]. Several reports have suggested that oxidative stress is involved in the pathogenesis of Mets [3,4], and oxidative stress levels are reported to be increased in human obesity and decreased with weight loss [5]. Oxidative stress is a condition in which reactive oxygen species (ROSs) are produced beyond their antioxidant capacity. Excessive amounts of ROSs that cannot be removed by the antioxidant system attack and damage proteins, lipids, and nucleic acids [6]; however, physiological levels of ROSs have essential roles in biological reactions [7].

AMP-activated protein kinase (AMPK) functions as a metabolic sensor and plays a vital role as a significant regulator in cellular energy homeostasis [8]. AMPK is implicated in metabolic syndrome, and its pathogenesis, insulin resistance [9,10], and ROSs are known to modulate AMPK signaling.

This study focused on the superoxide anion (O_2_·^−^) among the ROSs. O_2_·^−^ is produced by the leak of electrons under oxygen in the mitochondrial electron transport chain, xanthine oxidase, the cytochrome p450 monooxygenases, lipoxygenase, nitric oxide synthase, and the NADPH oxidase [11]. O_2_·^−^ is slowly dismutated to hydrogen peroxide (H_2_O_2_) and molecular oxygen spontaneously (rate constant = 8 × 10^4^/mol/s); however, superoxide dismutase (SOD) could rapidly dismutate O_2_·^−^ (rate constant = 2 × 10^9^/mol/s). The action of ROS may be working locally, and local kinetics should be considered [12]. Mitochondrial ROSs has been reported to activate AMPK secondary to altering the ADP/ATP ratio [13]. Therefore, we propose that O_2_·^−^ acts at the mitochondrial localization and contributes to metabolic syndrome’s pathogenesis via AMPK. Three types of SOD are known in mammalian cells. Among them, the manganese SOD (SOD2) present in the mitochondrial matrix and the copper-zinc SOD (SOD1) in the mitochondrial intermembrane space. SOD1 is also localized in the cytoplasm [11]. ATP sensed by AMPK is formed in the mitochondrial electron transfer system. The O_2_·^−^ formed by electrons leaking into the mitochondrial intermembrane space during this process is thought to be metabolized by SOD1. In this study, we investigated the role of SOD1 in the pathogenesis of Mets. Namely, systemic SOD1-deficient mice (SOD1^−/−^) were fed on a high-fat/high-sucrose diet (HFHSD) [14,15] and were compared with wild-type mice (WT).

## 2. Materials and Methods

### 2.1. Animal, Genotyping, and Diets

Male SOD1^−/−^ mice were obtained from Jackson Laboratory (Bar Harbor, Maine). These mice were backcrossed to C57BL/6J strain mice for eight generations as we did previously [16]. Next, we interbred heterozygous SOD1^+/−^ mice to obtain wild-type (WT) mice and homozygous SOD1^−/−^ mice within the same litter. Animals were housed in the facility on a 12-h light-dark cycle in a temperature-controlled room. The male SOD1^−/−^ and SOD1^+/+^ (WT) as control littermates were fed on either a standard diet (ND; CE-7; CLEA Japan, Inc. Tokyo, Japan) or a high-fat/high-sucrose diet (HFHSD; F2HFHSD with 28.3% of calories from carbohydrates, 54.5% from fat, and 17.2% from protein; Oriental Yeast Co., Ltd., Tokyo, Japan) ad libitum from 6 weeks of age for 24 weeks (Table 1). We made four groups (WT fed on ND, SOD1^−/−^ fed on ND, WT fed on HFHSD, SOD1^−/−^ fed on HFHSD). Body weights were recorded every two weeks. Genotyping for the SOD1 gene (accession number: NM_011434, XM_128337 and XM_358882) was performed by polymerase chain reaction analysis using genomic DNA isolated from the tail tip using the following primers.

WT 5′-TGAACCAGTTGTGTTGTCAGG-3′.

WT 5′- TCCATCACTGGTCACTAGCC-3′.

Mutant 5′- TGTTCTCCTCTTCCTCATCTCC-3′.

Mutant 5′- ACCCTTTCCAAATCCTCAGC-3′.

All experiments were conducted according to the institutional ethical guidelines for animal experiments and the safety guidelines for gene manipulation experiments of the National Defense Medical College. The experiments were approved by the Committee for Animal Research of the National Defense Medical College (approval number: 10063).

### 2.2. Tissue Preparation and Histology

Mice were anesthetized deeply with isoflurane and sacrificed. Blood was collected, and organs were perfused with Krebs’ Ringer bicarbonate solution (NaCl 118.3 mmol/L, KCl 4.7 mmol/L, CaCl_2_ 2.5 mmol/L, MgSO_4_ 1.2 mmol/L, KH_2_PO_4_ 1.2 mmol/L, NaHCO_3_ 25 mmol/L, D-glucose 5.5 mmol/L). The weight of liver, epididymal fat, and pancreas were measured, and liver, epididymal fat, aorta with fat tissue, and pancreas were fixed in 4% paraformaldehyde for 24 h, embedded in paraffin, and sectioned. These samples were stained with hematoxylin and eosin. Images were obtained with a microscopic system (BZ-X710; Keyence, Osaka, Japan). The cell size of adipocytes was measured with Image J.

### 2.3. Measurement of Metabolites

Serum levels of triglyceride, total cholesterol, and ALT were analyzed by enzymatic assays (FUJIFILM Wako Pure Chemical, Osaka, Japan). Blood glucose levels were determined using a blood glucose monitoring system, FreeStyle (Nipro, Osaka, Japan). Serum insulin levels were measured using ELISA kits (FUJIFILM Wako Shibayagi, Gunma, Japan). HOMA-IR as insulin resistance was calculated by multiplying fasting glucose level by fasting serum insulin level. An oral glucose tolerance test (OGTT) was performed after 12 h of fast. D-Glucose 3 g/kg body weight was orally administered. The vein blood was collected at 0, 15, 30, 60, and 120 min after glucose injection in OGTT.

### 2.4. Islet of Pancreas and Beta Cell

The weight of the pancreas was calculated from the weight of the β-cells using the method previously described [17,18]. The pancreas was stained with hematoxylin and eosin. Islet area per pancreatic area was calculated for three consecutive sections, and the average of the area percentage was defined as [islet area]/[pancreas area] (%). Then, double immunofluorescence staining of the pancreas was performed with both insulin and glucagon as beta cells and alpha cells of the islet, respectively. The tissue on the slides was permeabilized with 0.1% Triton X-100, and non-specific binding sites were blocked with 0.5% BSA. The slides were incubated overnight at 4 °C with both primary antibodies (anti-insulin antibody; 1:800 dilution; Cell Signaling Technology, Danvers, MA, USA; Cat#8138, anti-glucagon antibody; 1:400 dilution; Cell Signaling Technology; Cat#8233). After incubation, the slides were washed and incubated for 3 h at 37 °C with corresponding Alexa Fluor-488 (1:500 dilution; Cell Signaling Technology; Cat#4408) and Alexa Fluor-594 antibodies (1:250 dilution; Cell Signaling Technology; Cat#8889). The slides were washed three times and mounted under glass coverslips with Vectashield Antifade Mounting Medium containing DAPI for nuclear identification (Vector Laboraryories, Burlingame, CA, USA). Images were obtained with a BZ-X710 microscope (Keyence, Osaka, Japan). The area was calculated using the provided analyzing software. The area of each insulin- and glucagon-expressing cell was measured in three consecutive sections. The average of [insulin area]/[insulin area +glucagon area] was defined as [beta cell]/[islet area] (%). The weight of the beta cell was calculated by multiplying below.

[Weight of beta cell] = [Weight of pancreas] × [islet area]/[pancreas area] × [beta cell]/[islet area]

### 2.5. Oxygen Consumption and Food Intake

To assess oxygen consumption, carbon dioxide, and intake of food per day, each mouse was placed in each metabolic chamber system for 24 h after two days for getting (Columbus Instruments, Columbus, OH, USA). The food box with the same diet was kept in the metabolic chamber. The intake of food was calculated by subtraction of the weight after the experiment from the weight before. The diet and water were supplied ad libitum during the experiment.

### 2.6. Measurement of Skeletal Muscle and Vascular Superoxide Production

The superoxide production of aortic rings was assessed with dihydroethidium staining (DHE) (Thermo Fisher Scientific, Waltham, MA, USA), as previously described [19]. Skeletal muscles and aortic rings were snap-frozen with liquid nitrogen and embedded in an optimal cutting temperature medium (Sakura Finetek Japan, Tokyo, Japan). The frozen sections were immediately cut into 10-μm-thick sections and mounted on glass slides. Samples were then incubated at room temperature for 30 min with DHE (2 × 10^−6^ mol/L) and protected from light. Images were obtained with a microscopic system (BZ-X710; Keyence) with an excitation wavelength of 540 nm and an emission wavelength of 605 nm. The fluorescence intensity of DHE staining was measured using analyzing soft (Keyence).

### 2.7. ATP Level and ADP/ATP Ratio of Skeletal Muscle

Mice were anesthetized deeply with isoflurane and sacrificed. Immediately gastrocnemius muscles were collected and kept in liquid nitrogen. The muscles were homogenized with a proprietary buffer in the assay kit. ATP and ADP/ATP ratio was measured with a commercial kit, EnzyLight ATP assay kit, and ADP/ATP ratio assay kit (BioAssay Systems, Hayward, CA, USA).

### 2.8. Western Blotting of Skeletal Muscle and Epididymal Fat

Collected gastrocnemius muscles and epididymal fat were homogenized in homogenization buffer (Tris-HCl 20 mmol/L, pH 7.4, NaCl 150 mmol/L, Na_2_EDTA 1 mmol/L, EGTA 1 mmol/L, 1% NP-40, sodium pyrophosphate 2.5 mmol/L, monoglycerophosphate 1 mmol/L, Na_2_VO_4_ 1 mmol/L) containing phenylmethylsulfonyl fluoride (PMSF) 1 mmol/L. After centrifugation of homogenized samples at 13,000× *g* for 20 min at 4 °C, the supernatants as protein lysates were collected. Protein concentrations were measured by Bradford assay with standard using BSA [20]. Protein lysates were resolved by SDS-PAGE and transfer to PVDF membranes at 30 V for 2 h at 4 °C and immunoblotted with the primary antibodies to AMPK alpha (Cell Signal Technology Danvers, MA, USA; Cat#2532), phospho-AMPK alpha (Thr172) (Cell Signal Technology; Cat#2531), ACC (Cell Signal Technology; Cat#3662), phospho-ACC (Ser 79) (Cell Signal Technology; Cat#3661), SOD1 (Enzo Life Science, Farmingdale, NY, USA; Cat#ADI-SOD-101), SOD2 (Enzo Life Science; Cat#ADI-SOD-200), GAPDH (Cell Signal Technology; Cat#2118) and COX IV (Cell Signal Technology; Cat#4844).

### 2.9. Statistical Analysis

Results are shown as means ± SEM. The Kolmogorov–Smirnov test was used to test normality. Data for body weights, OGTT, and vascular relaxation were analyzed by 2-way ANOVA with repeated measures followed by the post hoc test with Bonferroni correction for multiple comparisons. The other data were analyzed by 1-way ANOVA followed by the post hoc test with Tukey’s multiple comparison procedure. All statistical analyses were performed with GraphPad Prism Software version 7 (GraphPad Software; La Jolla, CA, United States). Differences were considered significant if *p* < 0.05.

## 3. Results

### 3.1. Study Design and Changes with ND or HFHSD

**Figure 1:**
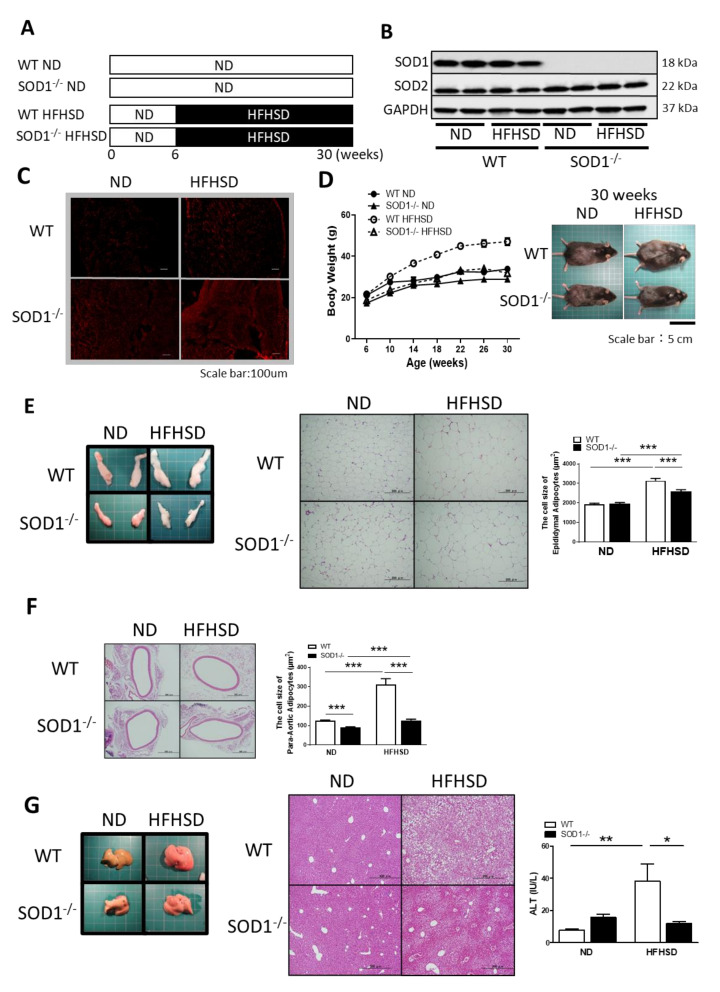
Study Design and Changes with ND or HFHSD. (**A**) Study design. HFHSD group fed with special diet for 24 weeks. (**B**) Western Blot Analysis of the skeletal. SOD1 deficiency was confirmed in SOD1^−/−^. (**C**) DHE staining of skeletal muscle. Red fluorescence indicates O_2_·^−^. Scale bar is 100 μm. (**D**) Weight curves for WT and SOD1^−/−^ on ND or HFHSD, respectively (WT on ND; *n* = 7, SOD1^−/−^ on ND; *n* = 7, WT on HFHSD; *n* = 6, SOD1^−/−^ on HFHSD; *n* = 9) and the picture of mice of WT and SOD1^−/−^ with ND or HFHSD after 24 weeks of HFHSD. The scale bar is 5 cm. (**E**) Appearance of epididymal fat, HE staining, and quantitative analysis of adipocyte size. The scale bar is 200 μm. (WT on ND; *n* = 4, SOD1^−/−^ on ND; *n* = 7, WT on HFHSD; *n* = 7, SOD1^−/−^ on HFHSD; *n* = 6). (**F**) HE stain of periaortic fat and quantitative analysis of adipocyte size. The scale bar is 500 μm. (WT on ND; *n* = 4, SOD1^−/−^ on ND; *n* = 7, WT on HFHSD; *n* = 7, SOD1^−/−^ on HFHSD; *n* = 6). (**G**) Appearance of the liver, HE stain and the level of serum ALT. The scale bar is 500 μm. (WT on ND; *n* = 16, SOD1^−/−^ on ND; *n* = 16, WT on HFHSD; *n* = 16, SOD1^−/−^ on HFHSD; *n* = 16). Error bars represent SEM. * *p* < 0.05, ** *p* < 0.01, *** *p* < 0.001. SOD: superoxide dismutase, DHE: dihydroethidium, HE: hematoxylin and eosin, WT: wild-type mice, SOD1^−/−^: SOD1 deficient mice, ND: normal diet, HFHSD: high-fat/high-sucrose diet, O_2_·^−^: superoxide anion, ALT: alanine aminotransferase.

WT and SOD1^−/−^ were divided into the normal diet (ND) and high-fat/high-sucrose diet (HFHSD) groups, respectively, for a four-group study; the HFHSD group received HFHSD for a total of 24 weeks from 6 to 30 weeks of age (Figure 1A). Western blot analysis of skeletal muscle showed similar expression of SOD2 but not SOD1 in SOD1^−/−^ compared to WT (Figure 1B). Dihydroethidium (DHE) (blue fluorescence) is converted to 2-hydroxyethidium (ex 500–530 nm/em 590–620 nm) (red fluorescence) by O_2_·^−^, so DHE staining was used to detect O_2_·^−^ in skeletal muscle. SOD1^−/−^ could not dismute O_2_·^−^, so red fluorescence was observed in the cytoplasm; in HFHSD, red fluorescence was enhanced even in WT, and the most robust red fluorescence was observed in SOD1^−/−^ fed on HFHSD (Figure 1C).

Weight gain of SOD1^−/−^ fed on HFHSD was blunted compared to WT and comparable to ND-fed mice despite the HFHSD (Figure 1D). To investigate the factors that contributed to the lack of weight gain in SOD1^−/−^, the degree of lipid storage in the organs was measured. Epididymal fat, liver, and periaortic fat were evaluated. The total size of epididymal fat from SOD1^−/−^ was smaller than WT fed on HFHSD, and the adipocyte size of SOD1^−/−^ was also smaller than WT in HFHSD, detected by hematoxylin and eosin (HE) stain (Figure 1E). Adipocyte size in periaortic fat was likewise smaller in SOD1^−/−^ than WT fed on HFHSD; even when treated with ND, it was significantly smaller in SOD1^−/−^ than WT (Figure 1F). HE staining of the liver showed fat deposition in HFHSD but to a lesser extent in SOD1^−/−^. Serum alanine transaminase (ALT) levels were also elevated in HFHSD WT, consistent with the findings of fatty liver. (Figure 1G). Serum triglycerides and total cholesterol were also lower in SOD1^−/−^ (Supplementary Figure S1). These results suggest that SOD1^−/−^ had less lipid storage than WT, even when fed on HFHSD, which may have contributed to the lack of weight gain.

### 3.2. Blood Glucose and Insulin Levels

**Figure 2:**
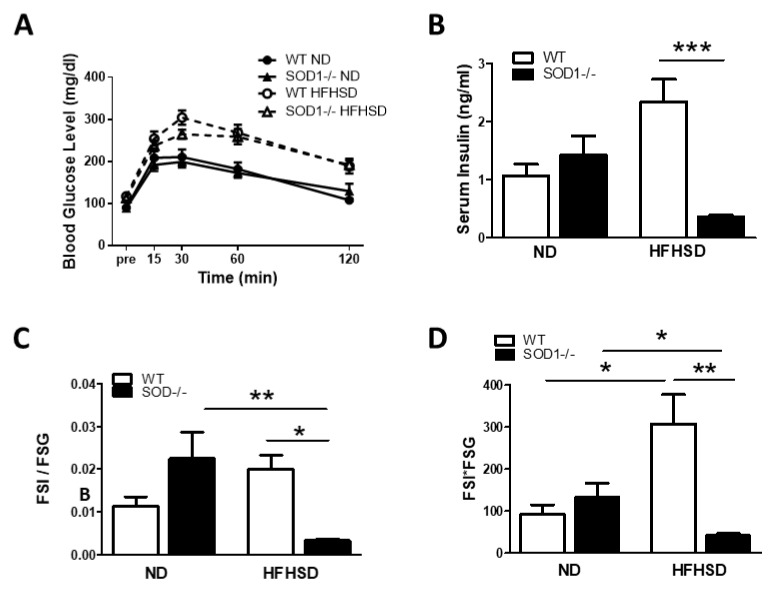
Blood glucose and insulin levels. (**A**). OGTT for WT and SOD1^−/−^ on ND or HFHSD at 30 weeks of age. (**B**). Serum insulin level in fasting state. (**C**). Blood glucose is divided by serum insulin level (HOMA-β equivalent). (**D**). The product of blood glucose and serum insulin levels (HOMA-IR equivalent). (WT on ND; *n* = 7, SOD1^−/−^ on ND; *n* = 8, WT on HFHSD; *n* = 10, SOD1^−/−^ on HFHSD; *n* = 8). Error bars represent SEM. * *p* < 0.05, ** *p* < 0.01, *** < 0.001. SOD: superoxide dismutase, WT: wild-type mice, SOD1^−/−^: SOD1 deficient mice, ND: normal diet, HFHSD: high-fat/high-sucrose diet, HOMA-β: homeostasis model assessment of beta cell function, HOMA-IR: homeostasis model assessment-insulin resistance.

Since lipid storage changes were observed in various organs, blood glucose and serum insulin levels were measured to determine insulin resistance. The OGTT (oral glucose tolerance test) showed similarly elevated blood glucose levels in both types of mice with HSHFD compared to ND (Figure 2A). On the other hand, serum insulin levels were lower in SOD1^−/−^ fed on HSHFD (Figure 2B). Serum insulin divided by blood glucose level (HOMA-β equivalent), indicative of insulin secretion, was significantly smaller in SOD1^−/−^ given HFHSD (Figure 2C). The product of blood glucose and serum insulin levels (HOMA-IR equivalent) indicates insulin resistance, and this index was elevated in WT given HFHSD; however, it was lower in SOD1^−/−^ given HFHSD (Figure 2D). In WT, HFHSD gained weight and increased insulin resistance, which increased blood glucose, whereas, in SOD1^−/−^, blood glucose was thought to increase due to decreased insulin secretion. The increase in blood glucose of SOD1^−/−^ fed on HFHSD was mild relative to decreased insulin.

### 3.3. Weight of Pancreatic β-Cells

**Figure 3:**
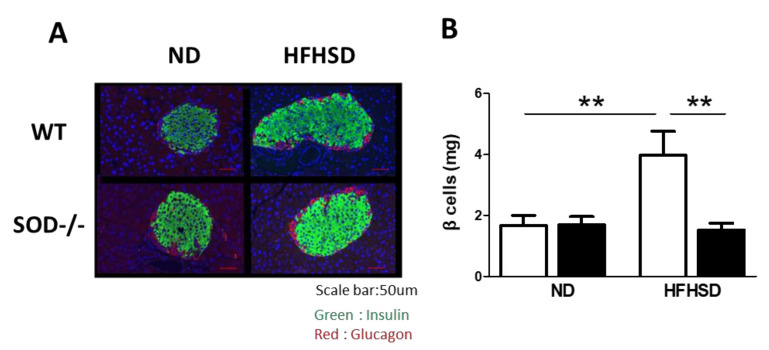
Weight of pancreatic β-cells. (**A**). Immunofluorescence staining of the pancreas with insulin (green) and glucagon (red) antibodies. The scale bar is 50 μm. (**B**). Quantitative analysis of pancreatic beta cell weights. (WT on ND; *n* = 8, SOD1^−/−^ on ND; *n* = 8, WT on HFHSD; *n* = 8, SOD1^−/−^ on HFHSD; *n* = 8). Error bars represent SEM. ** *p* < 0.01. WT: wild-type mice, SOD1^−/−^: SOD1 deficient mice, ND: normal diet, HFHSD: high-fat/high-sucrose diet.

Since insulin secretion was low in SOD1^−/−^, we examined morphological changes in the islets of the pancreas. The ratio of β-cells to islets was measured by immunofluorescence staining for insulin and glucagon (Figure 3A), and the weight of β-cells was estimated from the weight of the whole pancreas. WT on HFHSD increased pancreatic β-cells weight, but SOD1^−/−^ did not show any increase even on the HFHSD diet (Figure 3B). This result is consistent with the fact that serum insulin was not elevated in SOD1^−/−^ on HFHSD.

### 3.4. Oxygen Consumption and Intake of the Diet

**Figure 4:**
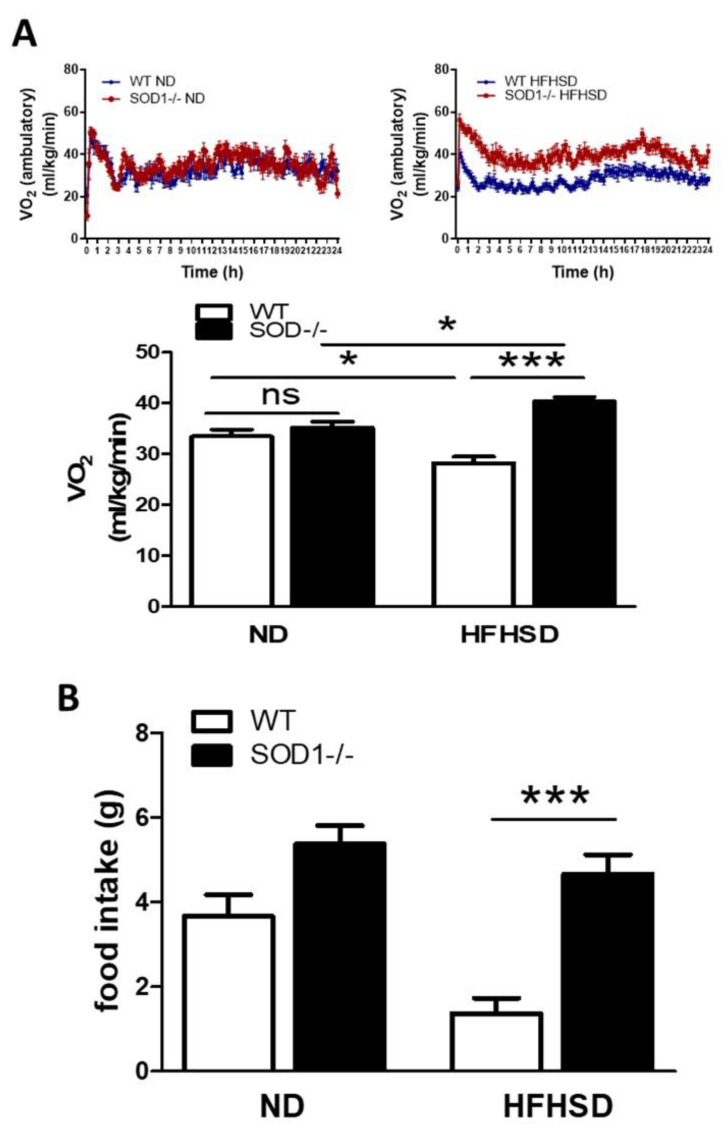
Oxygen Consumption and Intake of the Diet. (**A**). The oxygen consumption in ambulatory condition for 24 h and the quantitative analysis of averages oxygen consumption. (WT on ND; *n* = 7, SOD1^−/−^ on ND; *n* = 7, WT on HFHSD; *n* = 6, SOD1^−/−^ on HFHSD; *n* = 9). (**B**). Quantitative analysis of dietary intake. Error bars represent SEM. * *p* < 0.05, *** *p* < 0.001. WT: wild-type mice, SOD1^−/−^: SOD1 deficient mice, ND: normal diet, HFHSD: high-fat/high-sucrose diet.

Next, oxygen consumption was measured to examine the effects of insulin. Insulin is well known to induce to transfer and express GLUT4 to the cell membrane by attaching to the insulin receptor. Glucose transferred through GLUT4 is metabolized to Acetyl-CoA, NADH_2_^+^, and FADH_2_ is produced, and oxygen is consumed to produce ATP [21]. When insulin acts efficiently, oxygen consumption is thought to increase [22] and oxygen consumption is known to be depressed in insulin resistance [23,24,25]. Oxygen consumption was increased in SOD1^−/−^ fed on HFHSD compared to ND, whereas in WT, oxygen consumption was somewhat decreased in HFHSD compared to ND. (Figure 4A). The food intake was increased despite smaller body weight in SOD1^−/−^ with HFHSD (Figure 4B). Thyroid-stimulating hormone (TSH) and free thyroxine (free T4) levels did not differ among the four groups. Rectal temperature tended to be higher in the SOD1^−/−^ group, but there were no significant differences (Supplementary Figure S2). SOD1^−/−^ fed on HFHSD consumed more oxygen and food than WT, despite their smaller body weight. Insulin secretion was decreased in SOD1^−/−^, but the effect of insulin action was thought to be increased.

### 3.5. ATP Levels and AMPK Expression in Skeletal Muscle

**Figure 5:**
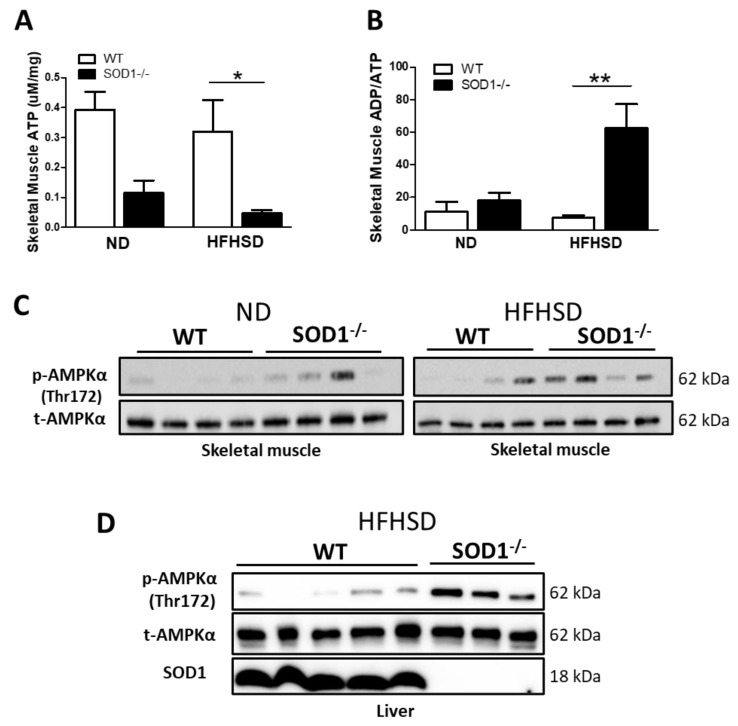
ATP levels and AMPK expression in skeletal muscle. ATP (**A**) and ADP/ATP ratio (**B**) of the skeletal muscle. (WT on ND; *n* = 8, SOD1^−/−^ on ND; *n* = 4, WT on HFHSD; *n* = 8, SOD1^−/−^ on HFHSD; *n* = 9). (**C**). Western blotting of skeletal muscle. (**D**). Western blotting of liver. Error bars represent SEM. * *p* < 0.05, ** *p* < 0.01. WT: wild-type mice, SOD1^−/−^: SOD1 deficient mice, ND: normal diet, HFHSD: high-fat/high-sucrose diet.

Mitochondria consume oxygen and produce ATP via oxidative phosphorylation and electron transport chain. Since SOD1^−/−^ fed on HFHSD consumed a lot of oxygen and diets, we hypothesized that there would be a lot of ATP in skeletal muscle. However, skeletal muscle ATP levels were lower in SOD1^−/−^ and significantly lower in the HFHSD compared to WT. ADP/ATP ratio was higher in SOD1^−/−^ with HFHSD (Figure 5A,B).

AMPK (AMP-activated protein kinase) monitors intracellular energy status and regulates glucose and lipid metabolism to maintain and restore energy balance. AMPK is activated by phosphorylation of Thr172, and the increase in AMP/ATP ratio and ADP/ATP ratio associated with the decrease in ATP inhibits the dephosphorylation of Thr172, leading to its activation. Then, we checked the phosphorylation of Thr172 of AMPK by Western blotting. Consistent with the decrease in ATP, phosphorylation of AMPK was observed in skeletal muscles of SOD1^−/−^ with HFHSD (Figure 5C). AMPK phosphorylation was also observed in adipocytes (data not shown) and liver (Figure 5D) in the HFHSD group of SOD1^−/−^.

## 4. Discussion

This study examined the role of O_2_·^−^ in metabolic syndrome in vivo. WT and SOD1^−/−^ were fed on HFHSD to develop metabolic syndrome [14,15] and compared with ND. SOD1 is present in the cytoplasmic and mitochondrial intermembrane space and degrades O_2_·^−^, which may alter the dynamics of O_2_·^−^ at that local site. Metabolic syndrome was induced by feeding on HFHSD [14,15] for 24 weeks, and as in previous reports [26], skeletal muscle from HFHSD-fed mice showed red fluorescence in DHE staining and even stronger fluorescence in SOD1^−/−^, reflecting cytosolic O_2_·^−^. SOD1^−/−^ had reduced weight gain and less fat deposition in HFHSD. WT showed insulin resistance and elevated blood glucose in HFHSD, while SOD1^−/−^ showed decreased insulin secretion and elevated blood glucose. However, the increase in blood glucose was mild compared to the decrease in insulin, suggesting that insulin was more effective in HFHSD-fed SOD1^−/−^. In WT, oxygen consumption was decreased in the HFHSD group compared to the ND group but instead increased in SOD1^−/−^. On the other hand, skeletal muscle ATP levels were lower in SOD1^−/−^ fed on HFHSD, with concomitant phosphorylation of AMPK Thr172. These results suggest that catabolism is enhanced in SOD1^−/−^ fed on HFHSD.

### 4.1. Body Weight, Blood Glucose and Insulin Changes

SOD1^−/−^ had less body weight than WT, even in ND; induction of metabolic syndrome in HFHSD resulted in attenuated weight gain, similar to WT in ND. From both clinical data and animal models, it is well known that insulin promotes anabolism and contributes to weight gain [27,28]. Fasting insulin concentrations tended to be lower in SOD1^−/−^ with HFHSD than ND, and insulin secretion in response to blood glucose was significantly reduced, contributing to the lack of weight gain and adipose mass. It has also been reported that SOD1^−/−^ alters the gastrointestinal microbiota [29] and may impair absorption. Furthermore, hydrogen peroxide is produced by SOD1 from the O_2_·^−^ and hydrogen peroxide is involved in cell hypertrophy [12]. SOD1^−/−^ was also claimed to be a model of sarcopenia showing various signs of aging [30]. These combined factors were thought to be responsible for the weight loss.

Blood glucose was elevated in SOD1^−/−^ with HFHSD but not high enough to account for the decrease in serum insulin levels. One mechanism for getting blood glucose into cells is GLUT4. Insulin activates AKT via insulin receptor substrate-1 (IRS-1) and phosphatidylinositol 3-kinase (PI3K), and AKT promotes GLUT4 translocation to the cell membrane [31,32]. Another regulator of GLUT4 is AMPK, which controls GLUT4 transport in an insulin-independent manner [33]. The reason why blood glucose was not higher in SOD1^−/−^ with HFHSD in the present study despite insulin levels may be that GLUT4 was upregulated by AMPK activation and the action of insulin was enhanced.

In WT with HFHSD, the estimated pancreatic β-cell weight increased, reflecting elevated serum insulin levels. In SOD1^−/−^ with HFHSD, insulin secretion was reduced; however, the estimated weight of β-cells remained the same and no morphological changes were observed. It was thought that the function of insulin secretion was impaired. Some studies showed that ROSs impaired insulin secretion [34,35]. On the other hand, there are reports that ROSs are involved in the mechanism of insulin resistance, and the mechanism was reported that chronic increase in ROSs led to phosphorylation of IRS-1 and decreased translocation of GLUT4 to the membrane [36]. In the present study, insulin resistance increased with increasing O_2_·^−^ in WT with HFHSD, while in SOD1^−/−^ with HFHSD, insulin secretion decreased and insulin resistance instead improved. This discrepancy may be due to the type of ROSs and their localization. SOD removes one of the ROSs (O_2_·^−^); however, at the same time, it produces another ROSs (hydrogen peroxide). The effects of ROSs are challenging to discuss in general, and the type of ROSs and their localization must be considered. Teratani et al. used SOD1^−/−^ and found that cytoplasmic hydrogen peroxide produced by SOD1 enhanced the amount and downstream signaling of DNA-bound PPARγ, leading to triglyceride accumulation in hepatocytes and liver [37].

### 4.2. Oxygen Consumption and AMPK Activation

It has been reported that mice on a high-fat diet consume less oxygen throughout the day than mice on a low-fat diet or a normal diet [23,24], and that fasting mice on the third day after starting a high-fat diet consume less oxygen than mice on a normal diet [25]. In this study, oxygen consumption was significantly reduced throughout the day in WTs with HFHSD. On the other hand, SOD1^−/−^ conversely increased oxygen consumption in HFHSD significantly. Dietary intake was significantly increased in SOD1^−/−^ with HFHSD, and body temperature tended to be higher in SOD1^−/−^, although there were no significant differences. The prominent place where oxygen is consumed is in Complex IV within the mitochondrial electron transfer system, where the electrons used to form the proton gradient to generate ATP combines with oxygen to form water. Contrary to expectations, however, ATP concentrations were relatively low in the skeletal muscle of SOD1^−/−^. Correspondingly, phosphorylated AMPK expression was elevated in SOD1^−/−^.

Regarding the relationship between AMPK and ROSs, while some reports suggest that exogenous hydrogen peroxide activates AMPK [38], others suggest it conversely inhibits its activation [39], and no sure view has been obtained. Hinchy et al. used O_2_·^−^ producing MitoParaquat in the mitochondrial matrix and reported that ROS from mitochondria does not directly activate cytosolic AMPK, but mitochondrial ROS reduces the ATP/ADP ratio and then activates AMPK [13]. Mitochondria are one of the sites of ROS production, and it has been reported that O_2_·^−^ is produced by electron leakage, which occurs at Complex I (reverse electron transport) [40] and Complex III [41]. O_2_·^−^ generated in Complex I are released into the mitochondrial matrix, whereas O_2_·^−^ generated in Complex III is released into both sides of the intermembrane [42]. SOD1 is present in the mitochondrial intermembrane space, and it is likely that the concentration of O_2_·^−^ in the mitochondrial intermembrane space is increased in SOD1^−/−^. The presence of O_2_·^−^ in the mitochondrial matrix and the mitochondrial intermembrane space may have reduced the ATP/ADP ratio, resulting in this result.

One of the possible mechanisms by which ATP is reduced in this study is the effect of uncoupling protein (UCP). UCP is a mitochondrial transporter protein that causes proton leakage across the mitochondrial inner membrane and functions to relax the proton gradient formed by the electron transfer system without producing ATP. The skeletal muscle used in this study is rich in UCP3. In addition to the insufficient scavenging capacity of O_2_·^−^ in the mitochondrial intermembrane space in SOD1^−/−^, HFHSD loading may have further increased O_2_·^−^. It has been reported that UCP is activated by superoxide anion s [43], and thus UCP3 in skeletal muscle may have been activated in SOD1^−/−^ fed on HFHSD. The increased oxygen consumption suggests that a proton gradient was formed by a large amount of oxygen in the electron transfer system; however, this gradient may have been attenuated by UCP3 without ATP production. Furthermore, it has been reported that UCP2 is present in pancreatic beta cells and that UCP2 is important for pancreatic beta cell dysfunction by inhibiting insulin secretion [44]. Another possible mechanism is that mitochondria-generated ROS may induce posttranslational modifications in ATP synthase, thereby modulating its activity [45].

In SOD1^−/−^, O_2_·^−^ in the mitochondrial intermembrane space were further increased by HFHSD loading, which may have activated UCP. In skeletal muscle, the decrease in ATP production due to UCP3 activation leads to increased oxygen consumption, and the increase in ADP/ATP leads to AMPK activation. Activated AMPK promotes cellular uptake of blood glucose via GLUT4 [46,47], and blood glucose levels did not increase significantly. In pancreatic beta cells, UCP2 prevents the production of ATP, which is necessary for insulin secretion, resulting in decreased insulin secretion and impaired anabolism, which may have led to decreased fat retention and weight loss.

## 5. Conclusions

O_2_·^−^ in the mitochondrial intermembrane space is metabolized by SOD1, but when accumulated, they inhibit ATP production, reduce insulin secretion, and promote metabolism in the catabolic direction. Proper metabolism of O_2_·^−^ in the mitochondrial intermembrane space is thought to lead to insulin resistance, which leads to the accumulation of visceral fat and the development of Mets.

## Figures and Tables

**Table 1:** Composition of Normal Diet and High-Fat/High-Sucrose Diet.

	Normal Diet		High-Fat/High-Sucrose Diet (F2HFHSD)	
	(CE-7)			
	g/100 g	kcal, %	g/100 g	kcal, %
Total calories	343 kcal		481 kcal	
Protein	17.7 g	20.6	20.7 g	17.2
Fat	3.8 g	10	29.1 g	54.5
Carbohydrate	59.4 g	69.4	34.0 g	28.3

## Data Availability

The data presented in this study are available in the article and Supplementary Materials.

## Supplementary Materials

The following are available online at https://www.mdpi.com/article/10.3390/antiox11071403/s1, Figure S1: Serum triglyceride and total cholesterol levels. Figure S2: Serum thyroid hormone test and rectal temperature.
