# Supplementary figures and images for: Resistance to Obesity in SOD1 Deficient Mice with a High-Fat/High-Sucrose Diet
Source: Antioxidants (Basel). 2022 Jul 19;11(7):1403. doi: 10.3390/antiox11071403 (PMC9312060; doi:10.3390/antiox11071403)

**A**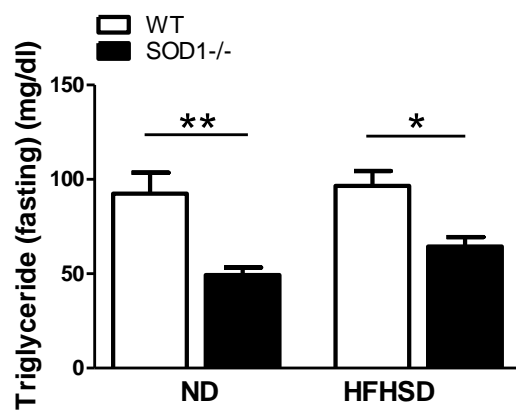**B**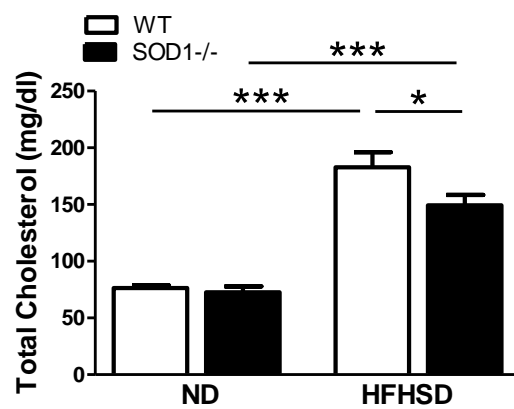

**A**

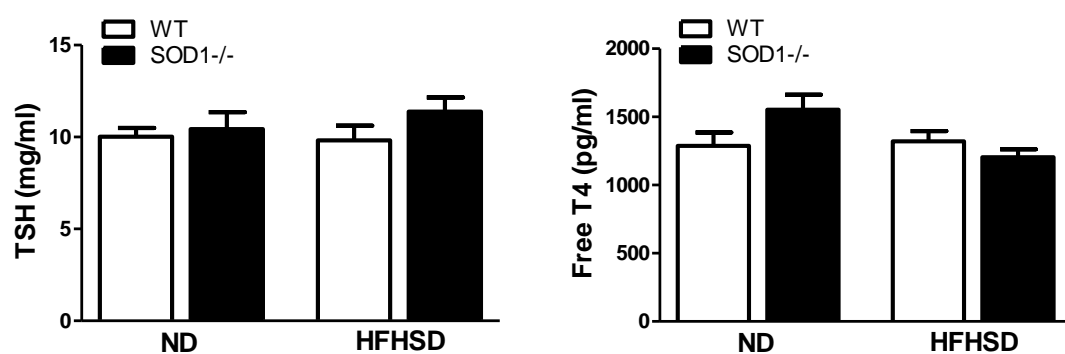

**B**

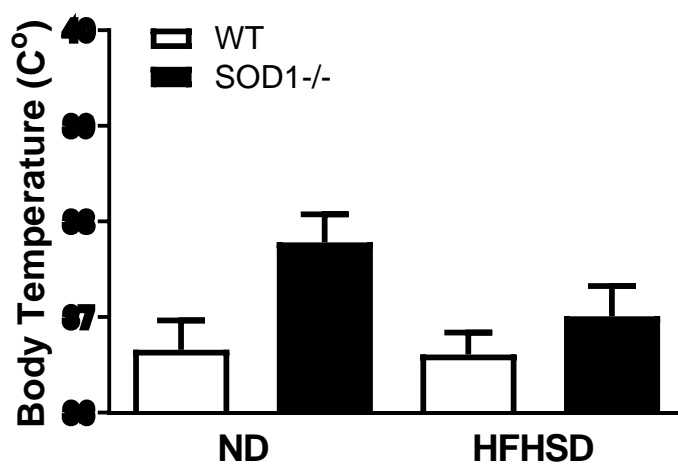

Supplement: Supplementary file 1 [file antioxidants-11-01403-s001.zip › antioxidants-1767454-supplementary.pdf]
